# Cambial activity and xylogenesis in stems of *Cedrus libani* A. Rich at different altitudes

**DOI:** 10.1186/s40529-015-0100-z

**Published:** 2015-07-28

**Authors:** Aylin Güney, Danielle Kerr, Ayça Sökücü, Reiner Zimmermann, Manfred Küppers

**Affiliations:** University of Hohenheim, Institute of Botany, Garbenstrasse 30, 70599 Stuttgart, Germany

**Keywords:** *Cedrus libani*, Cambium, Xylogenesis, Microcoring, Cell differentiation, Wood formation, Stem temperature, Traumatic resin ducts

## Abstract

**Background:**

The dynamics of cambial activity and xylogenesis provide information on how and to what extent wood formation respond to climatic variability. The Lebanon Cedar (*Cedrus libani* A.Rich) is a montane tree species which is distributed along a wide altitudinal range in the northeastern Mediterranean region, currently considered as a potential forest species for Central Europe with respect to climate change. This study provides first data on intra-annual growth dynamics at cellular level using the microcore technique for a montane Mediterranean tree species at different altitudes within and outside its natural range.

**Results:**

Microcores were collected fortnightly in the growing season of 2013 in order to study temporal dynamics of cambial activity and xylogenesis in stems of *C. libani* at different altitudes in the Taurus Mountains (1000 – 2000 m a.s.l.) and at a plantation at Bayreuth (330 m a.s.l.; Germany). The dormant cambium consisted of about 5 cells at the Turkish sites and 7 cells at Bayreuth. Cambial activity set in, when daily minimum temperatures exceeded 0 °C and daily means of air and stem temperature exceeded 5 °C. Xylogenesis started between April and May, ended approximately the end of September to the beginning of October and lasted 134 (at tree line) to 174 days (at the lowest Turkish site). Mean ring widths varied from 0.55 to 3.35 mm, with highest values observed at Bayreuth very likely resulting from a steady water supply during growing season. Means of daily cell production rates varied from 0.73 to 0.12. Samples containing traumatic resin ducts occurred only rarely and where not used for analysis.

**Conclusions:**

In *C. libani,* onset and dynamics of cambial activity and xylogenesis are triggered by daily means of stem and air temperatures whereas water availability has a higher influence on growth rates and cessation of wood formation. Within sites, duration of xylogenesis does not significantly differ with respect to age and tree size. *C. libani* grows well outside its natural range and thus may be a promising species for forestation in Central Europe with respect to climate change. We suggest further studies on if/how traumatic resin ducts influence tree ring width.

**Electronic supplementary material:**

The online version of this article (doi:10.1186/s40529-015-0100-z) contains supplementary material, which is available to authorized users.

## Background

Wood formation is a highly dynamic process regulated by physiological and environmental factors. It can be subdivided in onset, rate and duration of cell differentiation (Plomion et al. 2001; Deslauriers et al. 2003; Deslauriers and Morin 2005). Xylem cells are produced by the cambium and go through the phases of enlargement, secondary wall thickening, lignification and cell death (Plomion et al. 2001; Rossi et al. 2006c). Information on wood formation dynamics are necessary (a) to understand how and to what extent cambial activity and xylogenesis (with the different stages of cell development) respond to climatic variability and (b) to predict how forecasted climate change may influence tree growth and on the long hand forest productivity (Moser et al. 2009; Camarero et al. 2010; Vieira et al. 2014). Dendrochronological studies in general provide information on tree growth on an inter- annual to multi- centennial time scale and lack information as to when main events of tree ring formation take place within a single growing season. Therefore, intra- annual studies provide more detailed information on patterns and processes of xylogenesis within one growing season (Deslauriers et al. 2003, Rossi et al. 2007). The microcoring method (Rossi et al. 2006b) is easy to apply and allows to monitor the dynamic processes of cambial activity and xylogenesis. Some studies used the microcore method to investigate wood formation of Mediterranean tree species with the aim to determine the influence of climate parameters on cambial activity and xylogenesis. For example, Vieira et al. (2014) found that maritime pine cambial activity appears to be controlled by temperature (which triggers the onset of growth) and reduced water availability (which leads to cessation of growth). Camarero et al. (2010) analyzed intra-annual patterns of three conifer species from continental Mediterranean climates and showed that plasticity in xylogenesis patterns enables species to occupy sites with more variable climatic conditions. However, to our knowledge there are no studies present which investigate cambial activity and xylogenesis of a montane (oreophilous) Mediterranean tree species along an altitudinal gradient which could contribute to a better understanding on how the dynamics of wood formation change with altitude and varying site-specific conditions. Lebanon cedar (*Cedrus libani* A. Rich) is a drought tolerant conifer which is distributed along a wide altitudinal range (600 – 2300 m a.s.l) in Turkey, the Lebanon and Syria. Because of its stress tolerance and high timber quality many efforts in reforestation and afforestation have been undertaken within and outside its natural range (Boydak 2007; Boydak and Caliskan 2014; Fady et al. 2003; Khuri et al. 2000; Schütt et al. 2008; Uyar et al. 1990). *C. libani* appears to be a promising species to substitute tree species in Central Europe which might suffer from the expected climatic changes in their recent natural distribution areas. However, little is known about its growth dynamics. The objective of the study was to determine with the use of the microcore method (a) how cambial activity and xylogenesis in stems of *C. libani* take place along an altitudinal gradient (critical timings of the onset of cell production, termination of wood formation, cell number and ring width) (b) how dynamics of cambial activity and xylogenesis are linked with contemporary microclimatic conditions (air temperature, rain, drought) and (c) how xylogenesis of *C. libani* occur under central European climate conditions where a uniform water supply is available throughout the year (no severe summer drought). Based on the information of xylogenesis we present an initial insight on the effect of spring temperature on the onset of growth and the relative role of vegetation period on seasonal growth when drought periods are present or missing.

## Methods

### Study species

*Cedrus libani* A. Rich is an evergreen conifer of the *Pinaceae* family (Boydak and Calikoglu 2008).Today, *C. libani* has its largest distribution in Turkey with the purest stands located mainly between 800 and 2200 meters in the western Taurus Mountains where it reaches ages up to 1000 years (Dirik 2000; Akkemik 2003; Avci and Carus 2005; Carus and Avci 2005; Ducrey et al. 2008; Kurt et al. 2008). Stand sites are characterized by a summer dry season primarily between July and September. Annual mean temperatures range from 7.5 to 15 °C, with possible air temperature extremes as low as −35 °C and up to +40 °C (Aussenac 1984; Atalay 1987; Senitza 1989). Cedar forests contain a high amount of endemic species which make them biologically and ecologically valuable ecosystems (Kavgaci et al. 2010). Being highly adaptive to temperature extremes and summer drought, *C. libani* is one of the major commercial tree species in Turkey besides *Pinus brutia* and *Abies cilicica* (Brooks et al. 2008), and it consists of valuable wood which is very durable and versatile in application and use (Boydak 2003).

### Study sites

The study was conducted simultaneously in Turkey in the Southwestern Taurus Mountains at the Cedar Research Forest (CRF) near Elmali and in Germany at the Ecological-Botanical Gardens (EBG) at Bayreuth. Four study sites were chosen in the CRF along an altitudinal transect ranging from 1055 m to 1960 m a.s.l. and one at the EBG at 335 m a.s.l. (Table 1). Parts of the CRF that are forested mainly consist of pure *Cedrus libani* stands which range from 1000 m up to about 2200 m a.s.l., mixed occasionally with some *Juniperus* individuals. The transect stretches from the outer border of the forest near the Avlan lake to the upper timberline at 1990 m a.s.l. on a northwest-facing slope. The CRF is characterized by the Mediterranean mountain climate with relatively cold winters and a drought period during summer (from June to September) with a mean annual temperature of 7.3 °C (measured temperature extremes are −31 °C and +34 °C) and a mean annual precipitation of 644 mm (1970–2003, Turkish State Meteorological Service). The local climate in Bayreuth is subcontinental with a mean annual temperature of 7.9 °C (minimum: −25 °C, maximum: 36.1 °C) and mean annual precipitation of 724 mm (1971–2000; Foken et al. 2004). At the CRF clay and clay loam soils were dominant with pH varying from 7.2 to 8.1 (Basaran et al. 2008). At the EBG soil material was a mix of clay and sandy loam and pH varies from 4.4 to 5.3. At each site three individuals of *C. libani* with upright stems and no visible damages or parasitic infestations were selected (Table 1). *C. libani* individuals at the EBG and the CRF are of the same origin. All trees selected were adult and formation of juvenile wood was completed when sampling occurred (Bao et al. 2001; Plomion et al. 2001). Meteorological data such as stem temperature of the sampled trees and air temperature were recorded with thermocouples throughout the whole study period at each site respectively. Measurements were taken every 10 min and stored as hourly averages in data loggers (DL2e Data Logger, Delta-T Devices Ltd. Cambridge). For analysis, daily minimum, mean and maximum air temperatures as well as mean stem temperatures were calculated. Values of leaf area index (LAI) were obtained using the LAI-2000 plant canopy analyzer (Li-Cor, Inc., Lincoln, NE) at the Turkish sites (Table 1). Individuals at the EBG were planted in a single line from North to South with no significant shading, so LAI was not determined for this site. Precipitation data (monthly sums) of Bayreuth were derived from the meteorological station at the Ecological-Botanical Gardens of Bayreuth. For the Turkish site monthly sums of precipitation were derived from the meteorological station at Elmali (Turkish State Meteorological Service) which is situated circa 15 km afar of the CRF. At the sites T1 and T3 WatchDog weather stations (WatchDog 200 Series, Spectrum Technologies, Inc., IL, USA) were installed to measure precipitation with a 15 min frequency. Values were than computed as monthly sums. Due to data gaps at the measurement sites (T1 and T3) we plotted respective monthly sums of precipitation data available from the CRF with continuous precipitation data from Elmali to check if a linear relationship was present. Thereafter we performed nonlinear regressions (dynamic curve fit, polynomial, quadratic) using SigmaPlot (SigmaPlot software for Windows Version 10.0, Systat Software Inc., 2006) to compute and fill missing precipitation data at T1 and T3 (Fig. 3). An additional file shows the regression analysis we used to model missing precipitation data [see Additional file 1].

**Table 1:** Location of the study sites and characteristics of the sampled trees

ID	Site	CC	Latitude Longitude	Altitude (m a.s.l.)	Forest type	Population density (trees ha^−1^)	DBH (m)	Height (m)	Age at DBH (year)	LAI (m^2^ m^−2^)
T1	CRF Elmali	TR	36.58422°N	1960	Natural forest (Tree line)	478	0.6 ± 0.73	9.2 ± 3.7	42- 360 (152 ± 180)	0.95 ± 0.75
			30.03077°E							
T2	CRF Elmali	TR	36.58520°N	1665	Natural forest	501	0.39 ± 0.12	20 ± 2.1	84- 146 (112 ± 31)	4.7 ± 0.69
			30.02035°E							
T3	CRF Elmali	TR	36.57771°N	1355	Natural forest	533	0.4 ± 0.09	22.8 ± 1	90- 119 (101 ± 15)	1.49 ± 0.38
			29.98542°E							
T4	CRF Elmali	TR	36.57800°N	1055	Natural forest	334	0.44 ± 0.08	18.1 ± 3.5	65- 85 (75 ± 10)	2.0 ± 0.82
			29.96881°E							
O1	EBG Bayreuth	D	49.925915°N	335	Plantation	-	0.34 ± 0.03	12.3 ± 1.6	35	-
			11.582851°E							

The diameter at breast height (DBH), height of the sampled trees and the leaf area index (LAI) are presented as means with standard deviations (SD). The age of the sampled trees is shown as age range at DBH and as means with SD (in parentheses). At the site O1, all sampled trees were of same age, population density and LAI were not determined. ID: Site identification. CC: Country code. TR: Turkey. D: Germany

### Microcoring and histological analyses

**Fig. 1:**
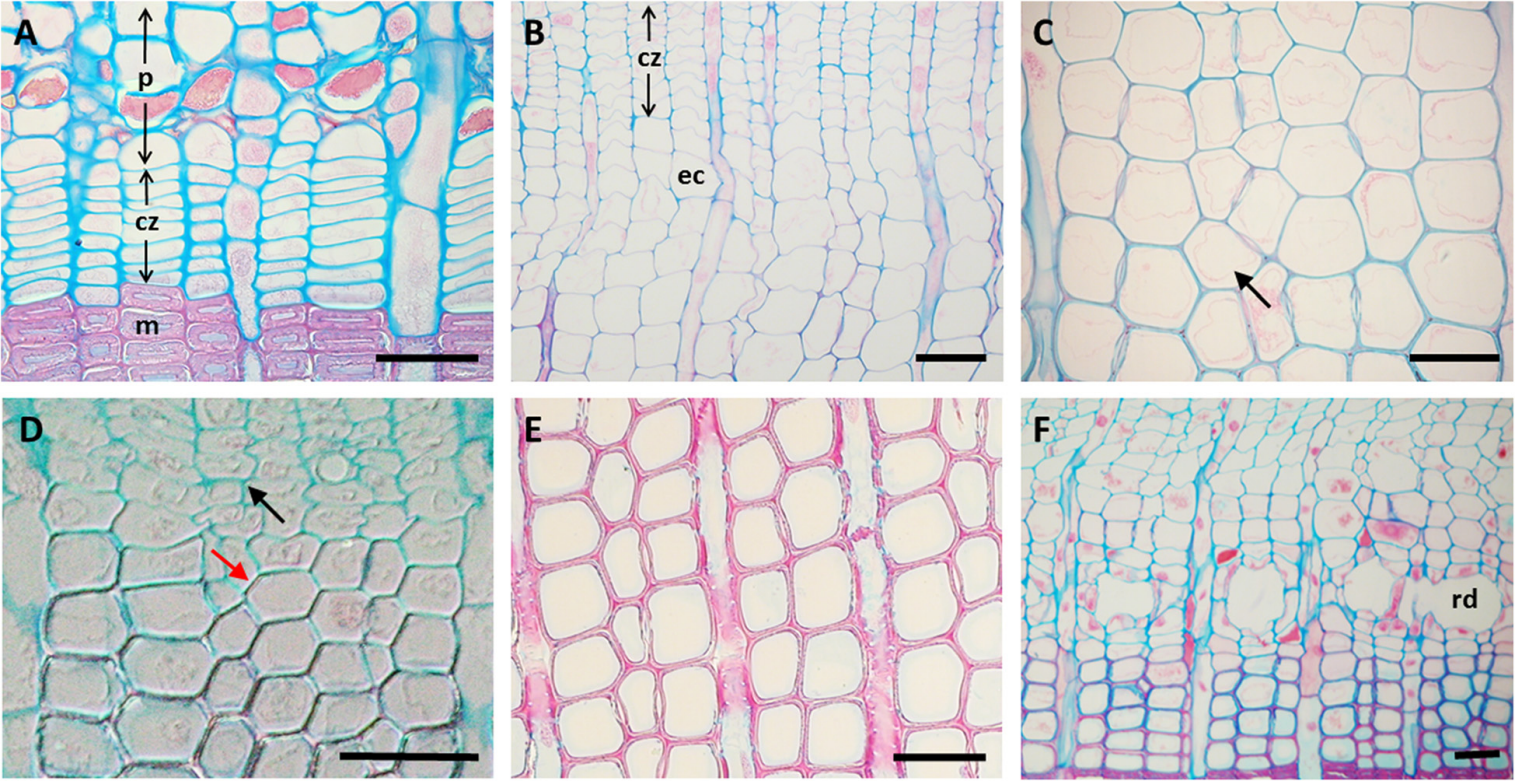
Cross sections of *Cedrus libani* stem wood*.* **a** Cambial zone (cz) during dormancy at the beginning of March with cambial initials and derivates, mature tracheids (m) of the previous year with thick secondary walls, and phloem (p) (**b**) Cambial zone during the growing season in June, enlarging cells (ec) (**c**) Wall thickening cells. The black arrow points at the intact protoplast of the cell (**d**) Enlarging cells which are not birefringent (black arrow) and wall thickening cells which are birefringent (red arrow) under polarized light (**e**) Mature cells with empty cell bodies and completely lignified secondary walls which appear intensely pink. **f** Resin ducts (rd) in the early wood during June. The bottom of the graphs are towards center of the stem. Scale bars: 50μm

Every 2 weeks during the growing season from March to October 2013, wood microcores were collected in each tree using a “Trephor” (Rossi et al. 2006b). The Trephor is a hand- sized tool with a cutting tube which is inserted into the wood using a rubber mallet. Samples were taken from stems at, as well as 50 cm below and above breast height (1.3 m) from the stem side parallel to the slope. Using a chisel, the outer bark was removed carefully before sampling at the sampling point was carried out. Microcore samples were 2 mm in diameter and between 5–20 mm in length, containing parts of the phloem, the cambium and the latest year rings of the xylem. To avoid tissue damage and formation of traumatic resin ducts (Fig. 1f) or other wood reactions, samples were taken following a spiral pattern along the stem surface leaving a minimum space of 5 cm between extraction points (Deslauriers et al. 2003). Immediately after sampling, microcores were placed in Eppendorf tubes (2 ml) containing a FAA fixative solution (formalin - acetic acid - alcohol, in a ratio of 5: 5: 90). To avoid tissue damage they were stored as soon as possible in a refrigerator until further use. After dehydration, using an ethanol series (70 % - 70 % - 90 % - 96 % - 100 % - 100 %; two hours steps), samples were embedded in glycolmethacrylate (Technovit 7100; Heraeus Kulzer, Wehrheim, Germany) with the transversal side facing upwards. Transverse sections were cut to a thickness of 3–5 μm using a rotary microtome (Microm HM 340 E), stained with fuchsine, safranin and astrablue (Etzold 1983) for about 45 min and fixed onto microscope slides with Roti (R) - Histokitt. In order to investigate cambial activity and wood formation dynamics, to distinguish cells in the cambial zone and to differentiate between cells according to their stage of xylogenesis (Fig. 1), transverse sections were examined and photographed on a Zeiss Axioplan microscope. The cambial zone consists of cambial initials and undifferentiated derivates with thin walls and small radial diameters (Fig. 1a). The onset of growth was determined by the first appearance of cells undergoing radial enlargement, resulting in cells larger than in the cambial zone and characterized by thin primary cell walls (Rossi et al. 2006a, 2007) (Fig. 1b). When radial expansion of tracheids ceased, secondary walls began to develop. In this so-called wall thickening stage cells still contained the protoplast (Fig. 1c) and started to appear birefringent under polarized light because of the special arrangement of cellulose micro fibrils in the secondary wall (Abe et al. 1997; Rossi et al. 2007) (Fig. 1d). Mature cells had empty cell bodies due to protoplast autolysis and completely lignified secondary walls (Fig. 1e). The end of growth was considered when all cells reached maturity (Rossi et al. 2006a, 2008b). In each sample the number of cell types were counted and widths of the actual performed tree ring were measured along three radial files and then averaged for subsequent analyses (Deslauriers et al. 2003; Rossi et al. 2006a, 2007; Thibeault-Martel et al. 2008). For each tree cell production rates were computed dividing the total number of cells formed within 2013 by the number of days spent in xylogenesis. Though it is not well known if and how much the presence of traumatic resin ducts influences wood formation and tree ring width we avoided samples with resin ducts in our study. In some sections where one part of the developing tree ring contained resin ducts and another part was free of them, it became clear that resin ducts generally did not affect the width of tree rings. However, at few occasions tree rings containing resin ducts appeared to be wider in radial direction as compared to parts without such ducts but studies with respect to this also show no clear pattern (Fahn et al. 1979; Wimmer and Grabner 1997).

### Statistical analysis

Univariate comparisons were used for means of cell number in dormant cambium, final number of xylem cells produced, daily cell production rate and annual tree ring width on the five sites. Homogeneity of variances among sites was assessed by Levene’s Test. In order to unveil whether xylogenesis (e.g. cell numbers, ring widths) was affected by site (altitude) we used Analysis of Variance (ANOVA). ANOVA and Tukey tests were performed for number of cells in dormant cambium when the requirements for homogeneity of variances were met, otherwise Welch’s ANOVA and Games- Howell tests were performed (for total number of xylem cells, cell production rate and ring width). Statistical analyses were performed using SPSS (IBM SPSS Statistics for Windows, Release 20.0.0, 2011)

## Results

From March to October 2013, calculated means of air temperature and stem temperature differed between all sites. Highest values were found at T4 with 16.9 °C for mean air temperature and 16.2 °C for mean stem temperature and lowest values at O1 with 11.7 °C and 12.3 °C, respectively. Mean air temperatures and mean stem temperatures were 15.3 °C and 15.1 °C for T3, 13.1 °C and 13.2 °C for T2 and 12.9 °C and 13.1 °C for T1. Precipitation was evenly distributed throughout the growing season at O1 whereas summer drought was present at the Turkish sites (Fig. 3).

**Fig. 2:**
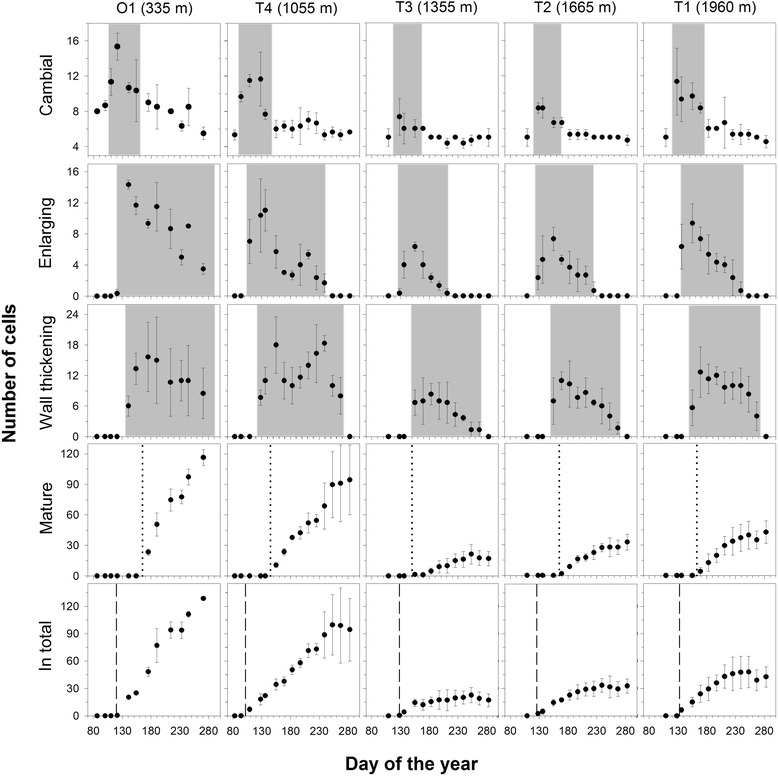
Timing and dynamics of cambial activity and xylogenesis in stems of *Cedrus libani* during 2013. Number of cells in the cambial zone, in radial enlargement phase, in wall thickening phase, mature cells and total number of xylem cells are shown for the German (O1) and the four Turkish sites (T1 –T4). Error bars represent standard deviations among trees per site. Periods of cambial, enlargement and wall thickening activity are highlighted in gray. Dotted vertical lines indicate the time when first mature cells could be observed, dashed vertical lines the time when enlarging cells emerged thus wood formation was initiated

During dormancy, when no cells have formed newly, the number of cells in the cambial zone (Fig. 1A) varied between four and nine (Table 2) showing significant differences among sites (ANOVA, F = 38.19, P < 0.001) with highest cell number at O1 (7.7 ± 1 cells; Tukey Test, P < 0.001). Among the Turkish sites (T4 to T1) cell numbers were similar and not significantly different (Tukey Test, P > 0.05). At all sites, the dormant period of the cambium lasted approximately from the beginning of August until April the following year. Cambial activity was first observed on April 3rd (day of the year, DOY: 94) at T4, followed by O1 (DOY 110) and the sites T3 to T1 (Fig. 2). Highest cambial activity was observed shortly after its initiation in spring and lasted 50 to 60 days. During this period the maximum number of cambial cells was observed at O1 (15 ± 2 cells).

**Table 2:** Timing of main events and characteristics of tree ring formation in stems of *Cedrus libani*

Site	O1	T4	T3	T2	T1
Observation	(335 m)	(1055 m)	(1355 m)	(1665 m)	(1960 m)
Number of cells in dormant cambial zone	7.7 ± 1.0	5.5 ± 0.5	4.9 ± 0.6	5.0 ± 0.5	5.0 ± 0.6
Onset of cambial activity	Apr 19 (110)	Apr 3 (94)	May 3 (124)	May 3 (124)	May 3 (124)
Onset of growth	Apr 30 (121)	Apr 10 (101)	May 3 (124)	May 3 (124)	May 15 (136)
Start of cell wall thickening	May 19 (140)	May 7 (128)	June 7 (155)	June 7 (155)	June 7 (155)
Dormant cambium	Sep 6 (250)	Aug 26 (239)	July 29 (211)	Aug 12 (225)	Aug 12 (225)
End of growth	Oct 10 (284)	Oct 1 (275)	Sep 24 (268)	Sep 26 (270)	Sep 26 (270)
Duration of cambial activity (days)	140	145	87	101	101
Duration of xylogenesis (days)	163	174	144	146	134
Total number of xylem cells	120.1 ± 8.5	96.6 ± 34.0	18.0 ± 6.4	31.6 ± 8.3	45.3 ± 13.4
Cell production rate (cells/day)	0.73 ± 0.05	0.55 ± 0.19	0.12 ± 0.04	0.21 ± 0.05	0.33 ± 0.1
Ring width (mm)	3.35 ± 0.65	2.76 ± 0.99	0.55 ± 0.19	1.01 ± 0.38	1.36 ± 0.39

For each site, number of cells in dormant cambial zone, radial growth, total number of xylem cells and cell production rate are presented as means with standard deviations. Numbers in parentheses indicate day of year (DOY) of first observation of the specified characteristics during the growing season 2013.

Once cells started to enlarge and the number of enlarging cells peaked in May and June, the number of cambial cells consequently decreased. Wall thickening was initiated approximately twenty days after the first cells reached the enlargement stage and it lasted for about 126 ± 17 days. Cambial activity, cell enlargement and wall thickening occurred sequentially forming a bell-shaped curve when the number of cells in a particular stage is plotted against time. Wall thickening was the stage that lasted the longest during xylogenesis (Fig. 2). The number of mature cells and the total number of cells formed at each sampling time point resulted in a sigmoidal curve (Fig. 2). When all cells had reached maturity and when no cells in the wall thickening stage were any longer present, xylem cell differentiation and thus growth of the year ring was considered to be complete. Growth ended first at T3 on 29th of July (DOY 211) and latest at O1 on October 10th (DOY 284). About four to five months were required to form a tree ring with the longest duration at T4 (174 days) and shortest at the highest site T1, with 134 days of xylogenesis (Table 2). For the means in total number of cells at the end of xylogenesis a high standard variation is obvious at T4 and T1, whereas it is low at O1, T3 and T2. Since within- group variances were not equal (Levene’s test, P < 0.01), a Welch’s ANOVA was performed, showing significant differences among sites (P < 0.001). The total number of xylem cells was significantly higher at O1 compared to T3, T2 and T1 (Games Howell Test, P < 0.001) and significantly higher at T4 compared to T3 and T2 (Games Howell Test, P < 0.05), while there were no significant differences comparing T3 and T2 (Games Howell Test, P > 0.05).

**Fig. 3:**
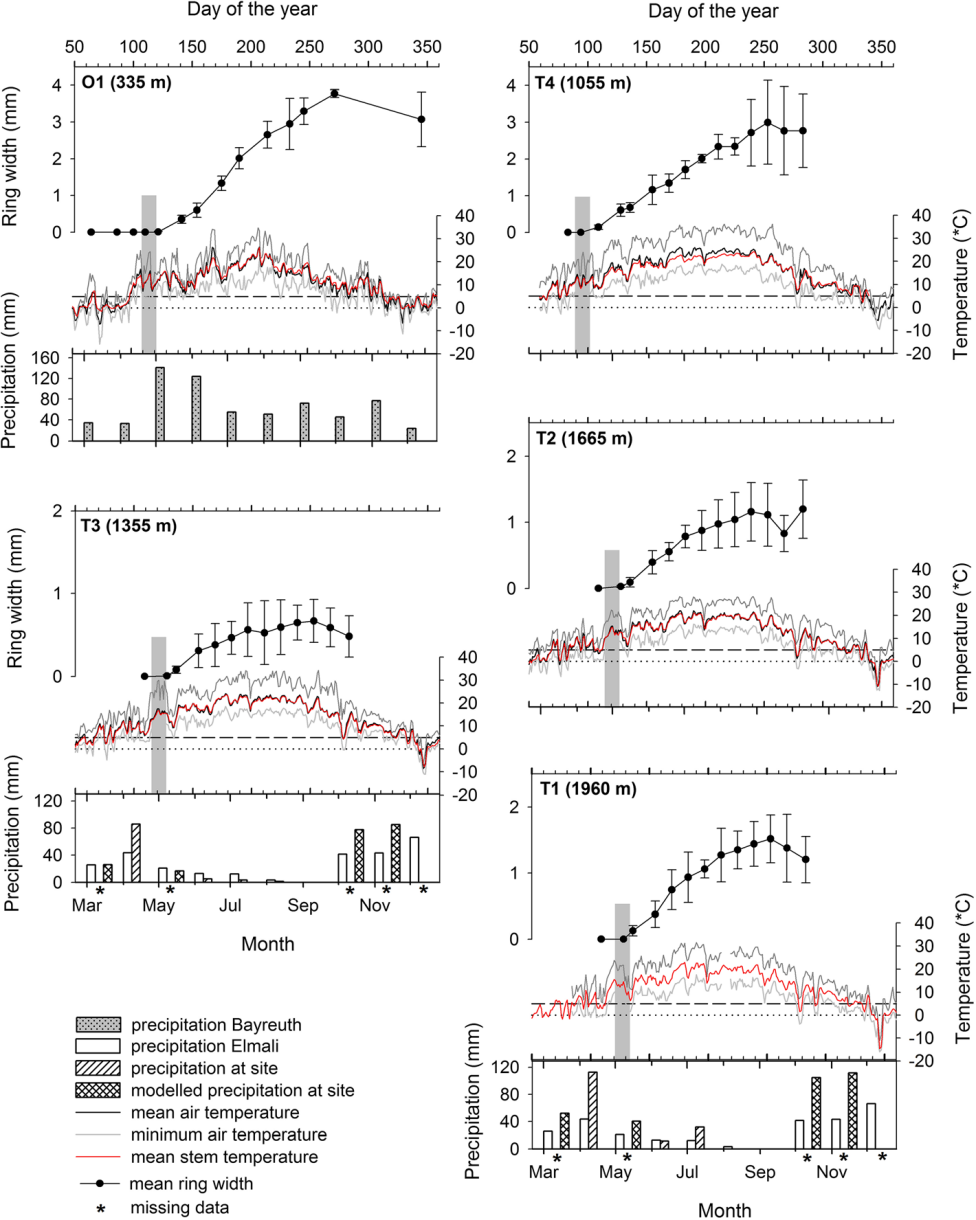
Tree ring formation in *Cedrus libani* with site specific microclimatic data. Shown are tree ring width resulting from radial growth with error bars indicating standard deviation among trees, daily maximum (dark gray line), mean (black line) and minimum (gray line) air temperature and daily mean stem temperature of sampled trees (red line) during the growing season of 2013. Vertical gray bands highlight the specific weather conditions over 14 days pre-running the first observation of enlarging cells and thus the beginning of radial increment. Precipitation data (monthly sum) are shown for O1 (grey, dotted bars), for T3 and T1. White bars show precipitation data from the Elmali Meteorological Station, hatched bars indicate precipitation data measured at the respective site, double-hatched bars show modelled precipitation data where site-specific precipitation data were missing (indicated with a star)

Mean values of daily cell production rates varied from 0.1 and 0.7 cells per day (Table 2, unequal within- group variances (per site); Welch’s ANOVA, P < 0.001). Cell production rates at O1 were significantly higher than at T3, T2 and T1 (Games Howell Test, P < 0.001) and significantly higher at T4 compared to T3 and T2 (Games Howell Test, P < 0.05), but did not differ significantly between sites T2 and T3 (Games Howell Test, P > 0.05). When measuring tree ring width from microcores, cells in the cambial zone were not included, so that tree ring formation started with the first presence of enlarging cells which is equivalent to the onset of growth (Table 2). When comparing it with weather conditions, it becomes evident that prior to the initiation of growth a pre- running fortnight free of frost is required (Fig. 3). From there onwards, daily means of air and stem temperature were above 5 °C and stayed above this level during the whole growing season.

Maximum radial growth occurred between May and July. Tree ring widths at the end of the growing season varied significantly among sites (Welch’s ANOVA, P < 0.001). Lowest ring widths at the end of growth occurred at T3 with 0.55 ± 0.19 mm and highest at O1 with 3.35 ± 0.65 mm (Table 2). Ring widths were significantly higher at O1 compared to T3, T2 and T1 (Games Howell Test, P < 0.001) and significantly higher at T4 compared to T3 and T2 (Games Howell Test, P < 0.05). The highest within-site variances regarding ring widths were observed at T4 with 2.76 ± 0.99 mm.

## Discussion

Several studies have been carried out regarding intra-annual wood formation in conifer and broadleaf trees (e.g. Camarero et al. 2010; Vavrcik et al. 2013), but information on how dynamics of wood formation occur in trees along an altitudinal gradient in the northeastern Mediterranean Mountains is completely missing. This study investigated cambial activity and xylogenesis in stems of *C. libani* along an altitudinal gradient in the Taurus Mts during the growing season of 2013. At the same time *C. libani* individuals were investigated at a plantation (EBG) under central European climate conditions where summer drought is largely missing. A clear relationship between temperature and cambial activity was obvious at all studied sites. Dynamics of xylogenesis differed between sites but showed no significant trend from low to high altitudes regarding duration of xylogenesis and the number of xylem cells formed during one vegetation period. Growth performance seemed to depend on water availability which was best at the German site. Number of cambial cells in the dormant cambium was significantly higher at the German site. Studies on other coniferous and broadleaf tree species (Gryc et al. 2011; Thibeault-Martel et al. 2008; Vavrcik et al. 2013) give similar number of dormant cells as observed at the Turkish sites (mean of 5 cells). Gričar et al. (2009)) report 3–11 cells in silver firs and a positive linear relationships between the number of dormant cambium cells and the total number of newly formed xylem cells. This leads to the conclusion that trees under good growth conditions are able to invest in a higher number of cambial cells which in return results in higher growth rates comparable with the results obtained in our study. This is especially noticeable with regard to the results from T4 (1055 m); here, duration of ring formation lasted longer than at O1 but annual ring width and the number of xylem cells formed at the end of the growing season were lower. In total, O1 showed best growth performance. The likely most important factor favoring growth at Bayreuth appears to be the absence of a drought period during summer and the uniform distribution of rainfall during the whole growing season. *C. libani* is a drought tolerant species (Ladjal et al. 2007), trees at the Turkish sites showed continuous growth during the growing season. However with respect to the results of other studies (Akkemik 2003; Touchan et al. 2005; Ducrey et al. 2008), tree ring width is positively correlated with the number of days with rainfall during the growing season and optimal growth performance is limited by lack of water during the growing season. This appears to cause significantly lower growth rates at the three upper Turkish sites, whereas because of its position close to the Avlan Lake, climate conditions at T4 may be more humid and temperate providing more favorable growth conditions within the Turkish sites. Many studies report that temperature is the main trigger for the onset of cambial activity and xylogenesis. It was observed in the Mediterranean species *Pinus pinaster* (Vieira et al. 2014) and especially in cold climates like boreal forests or tree line ecotones (Schmitt et al. 2004; Deslauriers and Morin 2005; Rossi et al. 2007). Threshold stem temperatures for the onset of growth are reported to be 7–9 °C (Rossi et al. 2007, 2008a; James et al. 1994). Investigations on Chinese pine (*Pinus tabulaeformis* Carr.) showed that cell division corresponded to the time when daily minimum temperature was above 0 °C (Liang et al. 2009). This is consistent with our observations in *C. libani*, where threshold temperatures for the onset of growth appeared to be 0 °C for daily minimum air temperatures and at least 5 °C for daily means of air and stem temperatures. When growth ceases in *C. libani*, lack of water seems to be the limiting factor while temperature would still favor growth. Vieira et al. (2014) reported that low water availability triggers pine wood formation in the Mediterranean area. Due to low water availability at the end of the drought period, growth ceases in *C. libani* at the end of September at the Turkish sites. For there is sufficient water supply at the German site, wood formation appears to cease when temperatures fall below a required minimum. When cambial activity finished, xylogenesis still continues with enlargement, wall thickening and lignification (Rossi et al. 2007). Our results clearly show that a lower number of cells in the dormant cambium as well as a low and short cambial activity result in a short growth duration and a small tree ring with only a small number of newly formed cells. According to the investigation of Rossi et al. (2007) at high altitude in the eastern Italian Alps on larch, stone pine and Norway spruce, duration of overall wood formation varies between 90 to 140 days. With time spans varying from 134 to 174 days, wood formation in *C. libani* lasted obviously longer. Observed differences in the duration of wood formation resulted mainly from varying timings in the onset of growth.

Sampled trees at our sites varied largely in age, but duration of cambial activity and xylogenesis did not significantly differ regardless of tree age and tree size within sites. When comparing the youngest (42 years) and the oldest tree (360 years) at the highest site T1 (1960 m), the latter showed a lower number of total cells formed during the growing season, but the onset, duration and end of growth appeared to be almost the same. This was observed at all sites, and this observation is also supported by hourly dendrometer measurements taken at the same sampled trees over the growing season (data not shown). These observations differ from results of Rossi et al. (2008a) who observed that timings and durations of xylogenesis differed between adult (50–80 years) and old conifers (200–350 years) at the alpine timberline, with a 2–3 weeks shorter cambial activity for the old conifers. It was also stated by various authors that the period of tree ring formation becomes longer the more cells are produced along a radial row (Gričar et al. 2005; Rossi et al. 2006b). This trend is in agreement with our Turkish sites. However, when comparing O1 and T4, it is fully evident that O1 produces significantly more xylem cells in an at least 10 day shorter growth period. The higher number of dormant cells therefore seems to host an important advantage for growth and allows a differentiation of more xylem cells in a shorter period. It can therefore not generally be concluded, that a longer period of ring formation leads to a higher number of cells. The highest number of cells in an active cambium was observed to be 17 at the German site and 15 at the Turkish site T4, which are much higher than observed by Thibeault-Martel et al. (2008) with 9 cambial cells in *Picea mariana* and 10–11 cells in *Abies balsamea*.

Along the altitudinal gradient, we expected a decreasing duration of cambial activity and xylogenesis from low to high sites because of decreasing temperatures. In fact, means of air and stem temperature during the growing season decreased significantly with altitude. But, while T4 (1055m) showed best growth performance as expected, the next site T3 (1355 m) showed lowest growth performance. Growth performance was significantly higher at T2 and much higher at T1 (tree line). Besides temperature and water availability this result could be affected by other parameters, such as stand density and light. T3 has the highest tree density, this combined with dryer conditions could result in the very low growth even though LAI was low. In comparison, tree density in T2 is lower than in T3 but the LAI showed the highest value for the Turkish sites. *C. libani* is a light demanding species (Senitza 1989). Among the upper three stands, the highest site becomes open towards the timberline, so light competition is the lowest here. Although the slope is north-west facing, due to the fact that it is located at the top of a mountain, it is exposed to the sun almost all day round. The same applies for O1, where trees are aligned in a single row orientated from North to South, and no significant shading occurs. The better light supply implies higher rates of photosynthesis which may in return positively influence growth performance. Sites T3 and T2 were more north- facing, denser in stand cover implying higher competition for light. Lower photosynthesis rates and stronger competition may be a reason for significantly lower growth performance at T3 and T2, but other reason that were not apparent (e.g. varying soil conditions within sites) may also be relevant, thus need further investigation.

## Conclusions

The xylem in stems of *C. libani* showed differences in onset, duration and end of cambial activity and xylogenesis as well as growth rates with respect to climate and site conditions. Temperature, especially daily means of air and stem temperature, appeared to be the main trigger when it comes to the onset of cambial activity and xylogenesis, whereas water availability and other factors, e.g. stand structure, light exposure etc., affect the total annual increment. Under the same site conditions (or within the respective sites) trees showed no significant differences in timing and duration of cambial activity and xylogenesis regardless of plant age and size. Cessation of growth is affected either by temperature or- as typical for the natural range habitats- by restricted water availability. Obviously, a temperate climate with an evenly distributed rainfall during the growing season is more favorable for *C. libani* than a Mediterranean climate. Our results therefore underline the potential of *C. libani* for afforestation outside its natural range with respect to climate change. Further studies on the relationship between traumatic resin ducts and tree ring width are necessary for a better understanding of the wood formation processes.

## Additional file

Additional file 1:
**Regression analysis for modelling missing precipitation data at the Turkish sites T1 and T3.** Available precipitation data of T1 (left box) and T3 (right box) were plotted against precipitation data from Elmali meteorological station (ca. 15 km afar of the Turkish sites). To estimate precipitation at the Turkish sites, available precipitation data from Elmali are inserted for x.

## Abbreviations

CRF: Cedar Research Forest
EBG: Ecological-Botanical Gardens
DBH: Diameter at breast height
DOY: Day of the year
LAI: Leaf area index
