# Supplementary figures and images for: Cambial activity and xylogenesis in stems of Cedrus libani A. Rich at different altitudes
Source: Bot Stud. 2015 Jul 28;56:20. doi: 10.1186/s40529-015-0100-z (PMC5430375; doi:10.1186/s40529-015-0100-z)

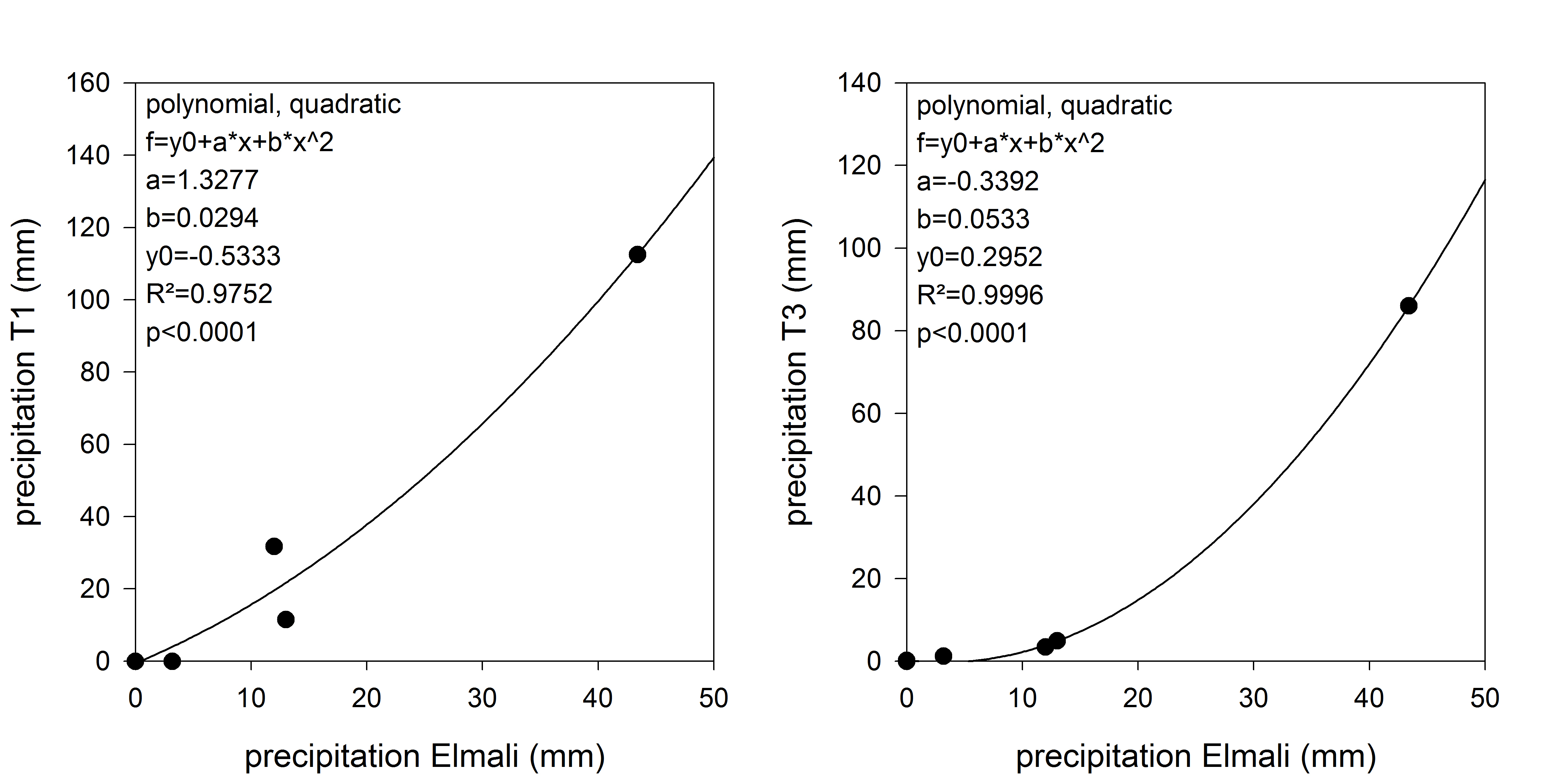

Supplement: Additional file 1: — Regression analysis for modelling missing precipitation data at the Turkish sites T1 and T3. Available precipitation data of T1 (left box) and T3 (right box) were plotted against precipitation data from Elmali meteorological station (ca. 15 km afar of the Turkish sites). To estimate precipitation at the Turkish sites, available precipitation data from Elmali are inserted for x. [file 40529_2015_100_MOESM1_ESM.jpeg]
